# In Vitro Evaluation and Comparative Analysis of Resorbable Membranes for Guided Bone Regeneration

**DOI:** 10.3390/medicina61091720

**Published:** 2025-09-22

**Authors:** Donato Antonacci, Rossella Padula, Federico Gaudelli, Irene Catalano, Filiberto Mastrangelo

**Affiliations:** Department of Clinical and Experimental Medicine, University of Foggia, 71122 Foggia, Italy; donat.antonacci@gmail.com (D.A.);

**Keywords:** guided bone regeneration, resorbable barrier membrane, hard tissues surgery, scanning electron microscope, EDX microanalysis

## Abstract

*Background and Objectives*: In vitro evaluation of macro and microscopic features of five resorbable barrier membranes used for Guided Bone Regeneration (GBR) in oral hard tissue surgery. *Materials and Methods*: Five different resorbable barrier membranes were analyzed by optical microscopy and scanning electron microscopy (SEM). For each sample, surface appearance, the presence and size of ridges and depressions, number of layers, and the inner structure were recorded. Each membrane was cut into 1 × 1 cm squares to determine mass, density and thickness. In addition, an EDX microanalysis was performed. *Results*: Under optical microscopy, all membranes appeared rough, with ridges and depressions. In cross-section, only Sample 2 presented true stratification. On SEM, most membranes showed a three-dimensional collagen fiber architecture. Sample 3, a sheet of collagenated equine bone, differed accordingly. EDX spectra showed broadly overlapping elemental composition, characterized by N, O and C. The mass depends on the composition: bone-containing membranes weighed more; those composed predominantly of collagen weighed less. *Conclusions*: Pore size, surface density and roughness, and the type of cross-linking can influence cell interaction and may lead to different regenerative scenarios, potentially improving the quality and timing of tissue regeneration. Membrane selection should be dictated by the clinical scenario, prioritizing properties most advantageous for the defect.

## 1. Introduction

In the era of digital workflow and guided surgeries, achieving adequate ridge volumes is a key determinant of clinical success [1].

Bone is a dynamic tissue: throughout life, it undergoes a continuous remodeling, with alternating cycles of resorption and deposition of new bone by osteoclasts and osteoblasts [2].

The post-extraction model is one of the most investigated because it is standardized, readily reproducible, and represents the most cause of maxillary bone defects. When a socket heals spontaneously, space closure occurs through the formation of granulation tissue [3,4].

Between 6 and 12 months after tooth loss, most of the decrease in alveolar ridge volume occurs: horizontal resorption can be reach up to 50% while vertical resorption varies from 11 to 22% [5]. This process is driven by the disappearance of periodontal structures such as the bundle bone and the capillary network related to the periodontal ligament, as well as the migration of soft tissues into the fresh socket.

These post-extraction changes have been documented in animal and human models. Bone loss is greater on the buccal side than on the palatal or lingual side [6], whereas mesio-distal levels show less resorption than the bucco-lingual/palatal dimension but worsen markedly with multiple extractions [7].

Another crucial factor is buccal/palatal plate thickness. Tomasi et al. (2010) demonstrated that resorption is much more pronounced when the plate is <1 mm thick and less so when thickness is >1 mm [8].

To mitigate this, alveolar ridge preservation (ARP) may be performed. When ARP is not possible or insufficient, subsequent augmentation is required [9].

In a reconstructed ridge, longer and wider implants can be used; moreover, an idealized ridge permits an adequate emergence profile that optimizes esthetics and facilitates hygiene [10].

Guided bone regeneration (GBR) is a regenerative technique that maintains space within a defect and mechanically prevents colonization by soft tissues, thereby allowing osteogenic cells to repopulate the site and regenerate lost bone. Epithelial exclusion is achieved with barrier membranes, while space maintenance is provided by bone particles, bone blocks, or natural/synthetic biomaterials [11,12,13,14].

Materials used for reconstruction of hard tissues include bone grafts, bone substitutes and barrier membranes. Barrier membranes serve as (i) a mechanical barrier to prevent migration and proliferation of connective-tissue cells from the soft tissues, thereby providing time and space for osteoprogenitor cells to colonize the socket and form new bone; and (ii) a space maintainer stabilizing the graft material. Although occlusive membranes effectively exclude epithelial cells, they may limit periosteal perfusion, making the regeneration dependent on basal bone blood supply [15]. Thus, membranes of greater thickness and density may provide an optimal barrier yet hinder perfusion from the periosteum.

Barrier membranes for GBR are broadly categorized as resorbable or non-resorbable. Non-resorbable membranes do not undergo enzymatic or microbial degradation. The most commonly used is expanded polytetrafluoroethylene (e-PTFE). Its external macroporosity promotes blood supply to the graft and allows extracellular components to spread, facilitating soft-tissue proliferation, while the occlusive internal structure prevents epithelial migration. Despite these advantages, non-resorbable membranes require a second procedure for removal and are associated with higher rates of dehiscence and infection.

Resorbable membranes, by contrast, are degraded enzymatically. Natural options are composed of type I and III collagen, whereas synthetic options use polymers such as polyurethane, polyglactin 910, polylactic acid, poly (ortho ester), polyethylene glycol, and polylactic-acid combinations. Resorption time varies with composition, pH, temperature, degree of polymer crystallization, cross-linking in collagen membranes, and membrane volume. In preclinical models, non-cross-linked collagen membranes showed advanced degradation 30 days after placement; blood vessels and bone matrix were detected within the membrane structure, indicating ongoing ossification [16]. Earlier studies reported 60% collagen degradation at 4 weeks and 80% at 9 weeks [17].

The barrier function should be maintained for at least 6 months to permit advanced osseointegration of the biomaterial [18]. Membrane use appears to increase the percentage of vital bone by limiting fibroblast ingrowth [19]. Accordingly, double-layer techniques or cross-linked collagen have been proposed [13]. A network meta-analysis on ARP underline how the crosslinked membrane performed better if not exposed due to their lower biocompatibility [9]. Cross-linking methods include aldehydes, ultraviolet exposure, hexamethylene diisocyanate, and diphenyl-phosphoryl azide. Reduced biocompatibility may impair neoangiogenesis and fibroblast/osteoblast chemotaxis [20,21]. Dehiscence or infection can jeopardize coronal ossification of the socket, reducing regeneration quality six-fold [22]. A systematic review discussed how cross-linking methods and morphology affect cell migration and proliferation [20]. An in vitro study reported greater gingival-fibroblast proliferation on native collagen membranes than on cross-linked ones [23]. In support, deproteinized bovine bone mineral (DBBM) and new bone were separated from cross-linked membranes by a thin collagen layer, whereas with non-cross-linked membranes, bone growth extended from the outer surface into the membrane, accompanied by capillary-network formation [24]. Similarly, when a native collagen membrane was interposed between the sinus mucosa and DBBM, the osteogenic properties of the sinus membrane were not hindered [25]. Recently, ribose-mediated cross-linking has been introduced as a “natural” method to increase collagen linkage without the biocompatibility penalty associated with aldehydes.

Overall, cross-linking, by itself, is not problematic; rather, the specific chemistry can render a membrane cytotoxic [20,26]. Conversely, slowing enzymatic degradation is desirable for successful bone regeneration and remains a key focus of tissue-engineering research. A double layer of non-cross-linked membrane may exploit high biocompatibility while prolonging the barrier effect to approximately 9 weeks; despite similar intrinsic resorption, patients receiving a double layer exhibited roughly double the residual collagen [17].

For this reason, resorbable barrier membranes in combination with a graft material represent the gold standard in GBR.

A wide range of GBR materials—and specifically barrier membranes—are available. Current research priorities include biocompatibility, bioactivity, porosity/occlusiveness, mechanical properties, exposure tolerance and biodegradability. Some authors have proposed combining collagen with perforated PTFE to optimize features, albeit at increased cost. Understanding how these properties arise requires detailed knowledge of membrane macro- and microstructure [27]. To our knowledge, no comparative study has comprehensively evaluated different membranes to identify characteristics that might make one more suitable than another for specific clinical scenarios. This in vitro study describes the macro- and microscopic differences among five resorbable barrier membranes used for GBR in dental hard-tissue surgery.

## 2. Materials and Methods

### 2.1. Optical and Scanning Electron Microscopy

Five different resorbable membranes currently available on the market were included in the study: Geistlich Bio-Gide^®^, OSSIX Plus^®^, Osteoxenon Flex^®^, XC Collagen Xenomatrix^®^, Straumann Membrane Flex^®^.

At the Microscopy Center of the University of L’Aquila, each membrane was analyzed under an optical microscope and with a SEM (Zeiss GeminiSEM500, Oberkochen, Germany) equipped with Energy Dispersive X-ray Spectroscopy (EDX) for chemical surface analysis performed at 20 kV for 1 min (OXFORD EDS AZtecLive ULTIM MAX 100, High Wycombe, UK).

Appropriate voltage SEM, in combination with the use of the Energy selective Backscattered (ESB) electron detector, was adopted to improve the optical contrast and mitigates the shrinkage of the membrane caused by electron beam-induced material damage. For each membrane, all SEM working conditions are reported at the bottom of the micrographs (WD—Working distance, Noise reduction frame average, EHT—Electron high tension, ESB—Energy selective backscattered, vacuum pressure). A ZEISS Axio Zoom.V16^®^ was used for optical microscopy and a ZEISS GeminiSEM^®^ for scanning electron microscopy. For each material, the surface appearance, the presence and size of reliefs and depressions, the presence or absence of stratigraphy in cross-section, and the three-dimensional microscopic morphology were described. Additionally, after fixing the samples in President^®^ adhesive paste, the cross-section was evaluated using optical microscopy.

### 2.2. Energy Dispersive X-Ray Spectroscopy

For each material, EDX microanalysis, also known as Energy Dispersive X-ray Spectroscopy (EDX), was also performed using the ZEISS GeminiSEM^®^. This technique measures the energy and intensity distribution of X-rays generated by the electron beam on the sample using an energy-dispersive detector. The technique allows for the identification of the chemical elements (elemental analysis) that make up the material or substance, even in trace amounts.

Finally, each barrier membrane was cut into square samples, 1 × 1 cm in size, and the mass and density were subsequently calculated.

### 2.3. Analysis

Each analysis was repeated on 6 samples of each membrane type investigated.

The data are presented descriptively, with mean values and standard deviations reported for the repeated measurements. In addition, the SEM evaluation is described qualitatively.

## 3. Results

### 3.1. Sample 1

#### 3.1.1. Commercial Information

Geistlich Bio-Gide^®^ is a native collagen membrane produced by a standardized, controlled process. Collagen is extracted from pigs with certified health status and is carefully purified to minimize immunological reactions. The membrane has a two-layer structure: the rough surface (bone-facing) promotes osteogenic-cell attachment, whereas the smoother surface (soft-tissue-facing) helps prevent fibrous tissue ingress. Sterilization is by gamma irradiation. The fibrous microstructure is hydrophilic, and the membrane retains structural integrity when wet. It is indicated as a barrier in bone-defect regeneration following bone grafting and to support soft-tissue formation. Owing to its adhesion to bone and elasticity, use with a bone graft is recommended. In the event of exposure during healing, absorption time may be accelerated. As with any collagen-based product, allergic reactions cannot be entirely excluded, and their physical characteristics and prolonged absorption time may be associated with inflammatory reactions. The membrane size is 25 × 25 mm.

#### 3.1.2. Optical Microscope and Scanning Electron Microscope Analysis

The optical microscopy analysis revealed the difference between the two surfaces, consistent with what is stated in the manufacturer’s leaflet. In fact, under a 5× magnification, it is possible to observe how the upper surface is smoother, as it is in contact with the soft tissues and serves as a barrier, while the lower surface is rougher, as it is in contact with the bone. Despite this, the upper surface also shows a microroughness, with an organized texture that follows a specific direction.

**Figure 1:**
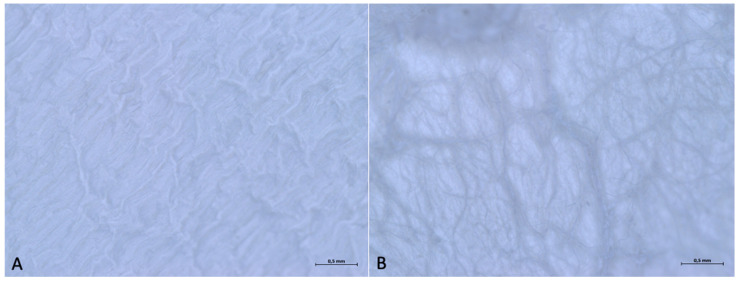
(**A**) Optical micrograph of the upper surface at 32× magnification. (**B**) Optical micrograph of the lower surface at 32× magnification.

As the magnification increases, even the upper surface, macroscopically smooth, has a rough texture, with depressions and dents (Figure 1).

**Figure 2:**
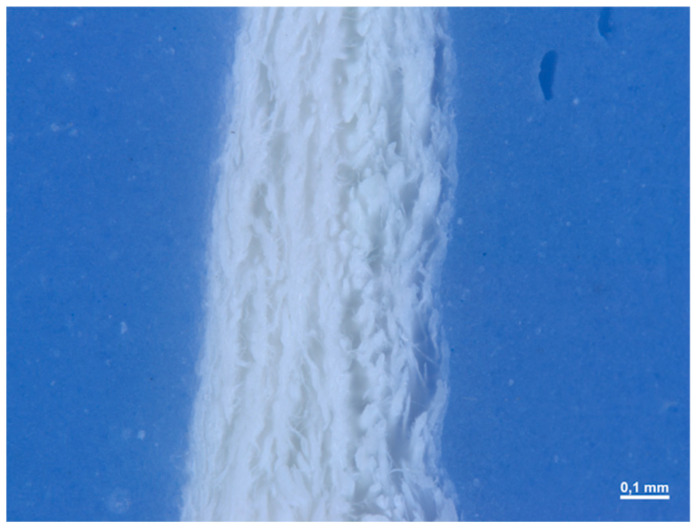
Cross-sectional image of the membrane at 100× magnification under the optical microscope.

In section, it is not possible to observe a precise stratification, since the material appears to be made up of an interwoven network of fibers without a precise organization. The mean thickness of the membranes is equal to 0.471 (0.021) mm (Figure 2).

**Figure 3:**
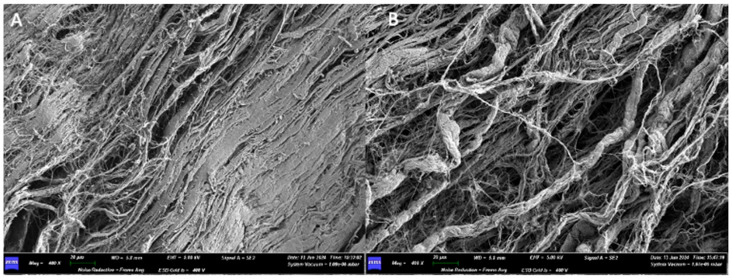
(**A**) SEM image of the upper surface at 400× magnification. (**B**) SEM image of the lower surface at 400× magnification.

Under the scanning electron microscope (SEM), with a 30× magnification, it is possible to appreciate the upper surface, smooth, has a certain roughness given by a texture that follows a certain orientation (from the lower left corner to the upper right). On the lower surface, one can appreciate the presence of a dense network of collagen fibers, which follow a random orientation (Figure 3).

At 400× magnification, the upper surface shows a high degree of compaction, making it smooth. On the lower surface, the collagen fibers can be observed, looser and less tightly packed than on the upper surface. Several fibers come together to form bundles. The widths of some fibers are 1250 µm, 8750 µm, and 10,000 µm.

**Figure 4:**
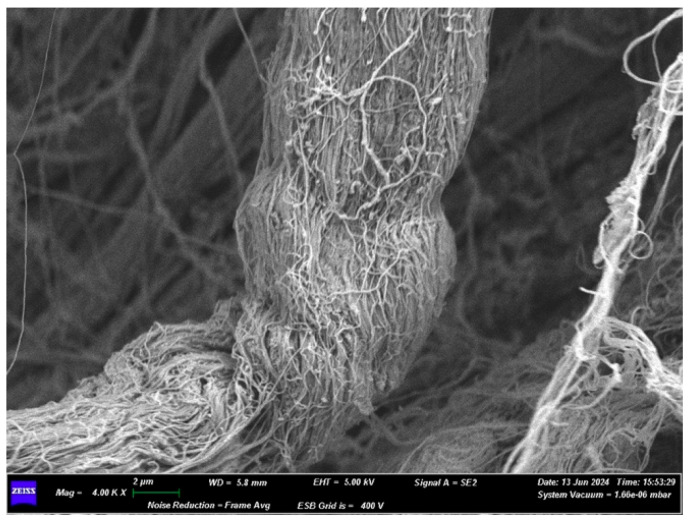
SEM image of the lower surface at 4000× magnification.

At a 4.00K× magnification of the lower surface (Figure 4), a bundle of collagen fibers can be observed. Measurements taken at multiple points revealed the following bundle widths: 7429 µm, 6190 µm, 7238 µm. The widths of some individual fibers are 0.095 µm and 0.190 µm.

**Figure 5:**
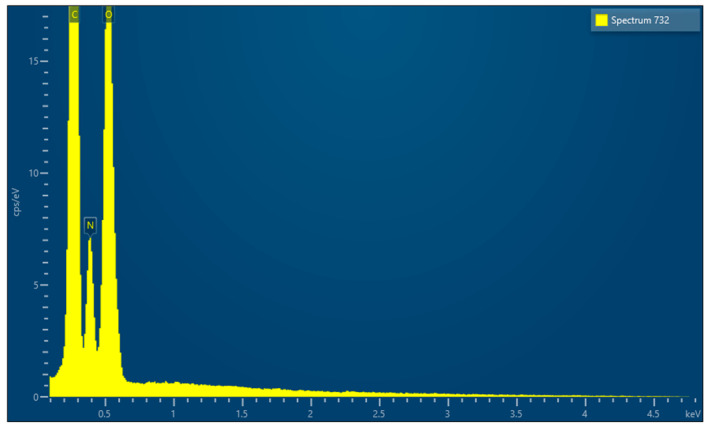
EDX results.

EDX microanalysis performed by scanning electron microscope showed the chemical elements constituting the membrane: C, N and O (Figure 5).

Finally, after cutting the membranes into a square-shaped sample measuring 1 cm × 1 cm, the mass of the sample was calculated to be 9.7 (0.2) mg. After determining the volume of the samples (V = a × b × c = 1 cm × 1 cm × 0.0471 cm = 0.0471 cm^3^), we calculated their main density (d = m/V), which is 0.206 (0.02) g/cm^3^. Very slight differences were found between the membranes in terms of microtopography or thickness.

### 3.2. Sample 2

#### 3.2.1. Commercial Information

Commercial information OSSIX^®^ Plus is a biodegradable collagen membrane extracted from porcine tendons and purified to minimize hypersensitivity. It is supplied in a double-sealed blister and sterilized by ethylene oxide. It exhibits excellent biocompatibility, a porous structure designed to block gingival cells while permitting fluids and plasma proteins—thereby facilitating flap closure. As it is not self-supporting, use with a bone graft is recommended. The membrane maintains integrity when wet and adapts readily to the alveolar ridge. An animal study reported complete degradation at approximately 8 months. Outcomes may be compromised in high-risk patients (e.g., smokers, uncontrolled diabetes mellitus, uncontrolled periodontal disease). The membrane analyzed measured 15 × 25 mm.

#### 3.2.2. Optical Microscope and Scanning Electron Microscope Analysis

The optical microscope examination did not reveal any differences between the two surfaces of the membrane. At 16× magnification, it is possible to observe that both sides feature an organized pattern made up of oval geometric shapes of two types (one larger, elongated in the vertical direction, and one smaller, elongated in the horizontal direction). The geometric pattern appears to have a specific direction, extending from the top-left corner to the bottom-right corner.

**Figure 6:**
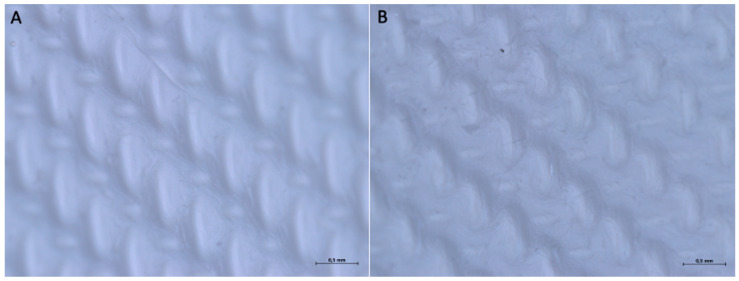
(**A**) Optical micrograph of the upper surface at 32× magnification. (**B**) Optical micrograph of the lower surface at 32× magnification.

With a 32× magnification, on surface A, it is possible to see that the ovoid shapes elongated in the vertical direction have dimensions of approximately 0.114 × 0.636 mm, and the ovoid shapes elongated in the horizontal direction are 0.227 × 0.114 mm. On Surface B, the ovoid shapes elongated in the vertical direction have dimensions of approximately 0.227 × 0.568 mm and the ovoid shapes elongated in the horizontal direction 0.295 × 0.114 mm (Figure 6).

**Figure 7:**
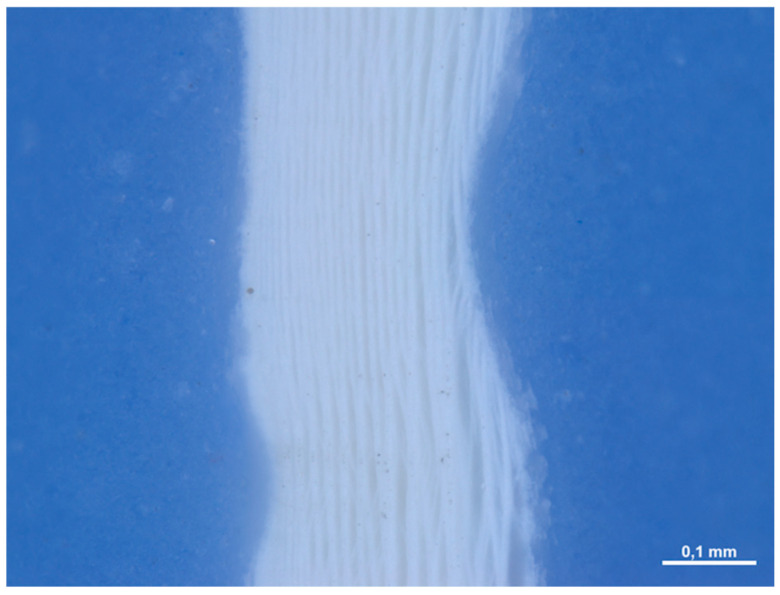
Cross-sectional image of the membrane at 168× magnification under the optical microscope.

In section, the membrane appears to be made up of multiple layers pressed and compacted until a single sheet is obtained (Figure 7). The average thickness of the membrane is approximately 0.245 (0.014) mm.

Under the scanning electron microscope (SEM), with a 400× magnification, it is possible to observe how the two surfaces have no structural differences. We can appreciate the presence of a dense network of collagen fibers, but with different areas of compaction: in the areas where the pressing has left the imprint, the collagen fibers are less visible, while in the other areas they are looser and more appreciable.

Increasing the magnification, a dense network of collagen fibers can be observed, interwoven with each other in a completely random pattern, without following any defined structure. The width of some fibers is reported as follows: on side A, 0.100 μm, 0.150 μm, 0.200 μm; on side B, 0.050 μm, 0.100 μm, 0.150 μm, 0.300 μm.

**Figure 8:**
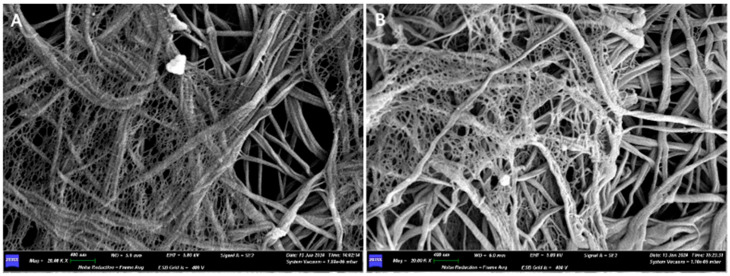
(**A**) SEM image of the upper surface at 20,000× magnification. (**B**) SEM image of the lower surface at 20,000× magnification.

Only at high magnifications (20.00K×), on both sides of the membrane, is it possible to see a micro-network of fibers that envelops and supports the collagen fiber bundles in certain areas (Figure 8).

**Figure 9:**
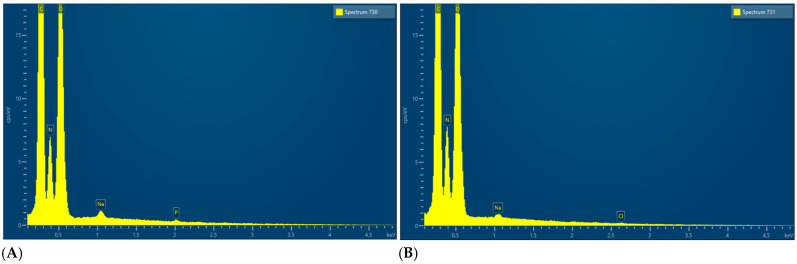
(**A**) EDX results of site A. (**B**) EDX results of site B.

EDX microanalysis, performed on two different sites of the membrane using the scanning electron microscope, showed the chemical elements constituting the membrane: C, N, O, Na, Cl and P (Figure 9).

Finally, after cutting the membranes to obtain a square-shaped sample, measuring 1 cm × 1 cm, the mass of the sample was calculated, which is equal to 6.6 (0.13) mg. After obtaining the volume of the samples (V = a × b × c = 1 cm × 1 cm × 0.0245 cm = 0.0245 cm^3^), we calculated their main density (d = m/V), which is equal to 0.269 (0.04) g/cm^3^.

Almost no differences were found between different membranes from the same sample.

### 3.3. Sample 3

#### 3.3.1. Commercial Information

Commercial information Osteoxenon^®^ Flex Cortical Sheet (OSP–OX09) is a collagenated, deantigenated and partially demineralised equine bone sheet. The barrier effect lasts at least 6 months, after which occlusiveness is lost owing to osteoclastic resorption. It may be used alone or with appropriate grafting materials for GBR. A mandatory traction direction, indicated by an edge indentation, must be respected; traction forces should be applied parallel to this. If needed, shape the membrane before hydrating; hydrate in sterile saline for 1–2 min. Sterilization is by beta irradiation at 25 kGy. Sizes available: 21–25 × 23–27 × 0.2 mm (OSP–OX03), 21–25 × 23–27 × 0.5 mm (OSP–OX09), and 21–25 × 23–27 × 0.8 mm (OSP–OX08). The membrane studied was OSP–OX09.

#### 3.3.2. Optical Microscope and Scanning Electron Microscope Analysis

When examined under an optical microscope, at 16× magnification, it can be seen that both surfaces are rough but follow a different pattern. One surface (surface A) has a regular texture, with parallel horizontal striations, which create a striped effect, while the other (Surface B) has a roughness generated by depressions of variable shape and scattered irregularly over the entire surface. Both sides of the membranes have dark dots scattered irregularly over the entire surface.

**Figure 10:**
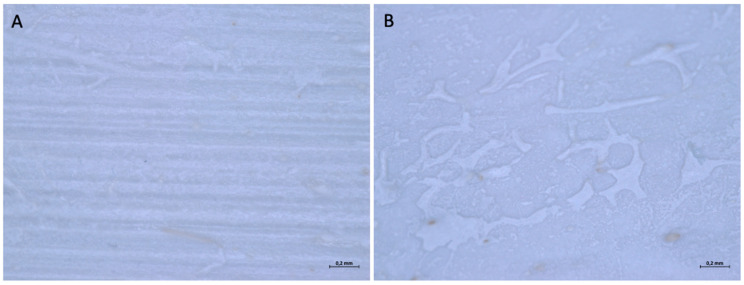
(**A**) Surface A observed under an optical microscope at 56× magnification. (**B**) Surface B observed under an optical microscope at 56× magnification.

With a higher magnification, 56×, it is possible to better appreciate the textures that constitute the two sides of the membrane (Figure 10). On surface A, the distance between one stria and the other has a variable dimension; some values are reported to be 0.042 mm, 0.021 mm, and 0.053 mm.

**Figure 11:**
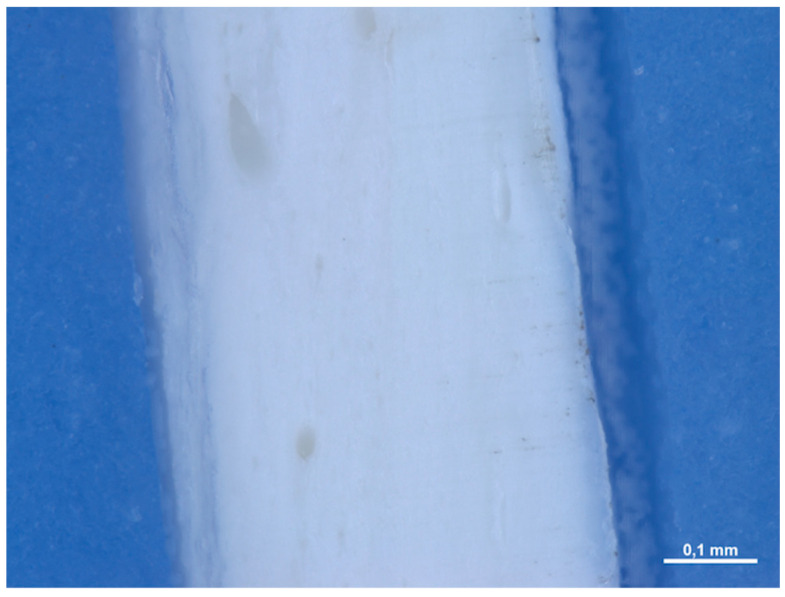
Cross-sectional image of the membrane at 168× magnification under the optical microscope.

In section, the membrane has a compact structure, with the presence of some porosity in some points. The pores have a variable shape: some are rounder, others are elongated (Figure 11). The main thickness of the membranes is equal to 0.436 (0.044) mm.

When examined with a scanning electron microscope (SEM), at a magnification of 200×, it is possible to appreciate how the structural difference between the two sides of the membrane observed with the optical microscope is also evident at the microscopic level. In fact, side A is more regular, has a compact surface with horizontally parallel striations and the presence of pores. The diameter of the pores is variable: 0.055 mm, 0.012 mm, 0.035 mm, 0.01 mm, 0.005 mm. Surface B is more irregular, with roughness generated by pores and depression zones of variable shape scattered over the entire surface in an irregular manner. The diameter of the pores is also variable here: 0.025 mm, 0.017 mm, 0.050 mm.

With a magnification of 1.50K×, it is easier to see that Surface A appears smoother and more regular, with the presence of pores, while Surface B has a dense network of micro and macro pores.

**Figure 12:**
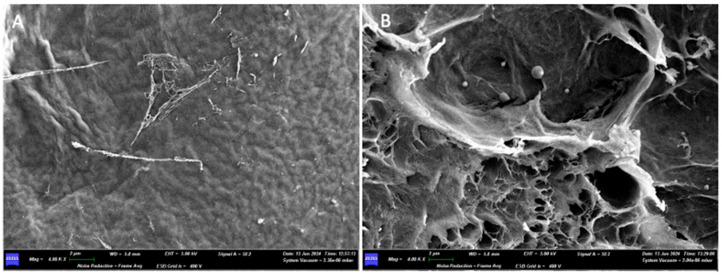
(**A**) Surface A observed under SEM at 4000× magnification. (**B**) Surface B observed under SEM at 4000× magnification.

Going up further with the magnifications, with a 4.00K× it is possible to see how on both sides of the membrane there are collagen fibers adhered to the two surfaces (Figure 12).

**Figure 13:**
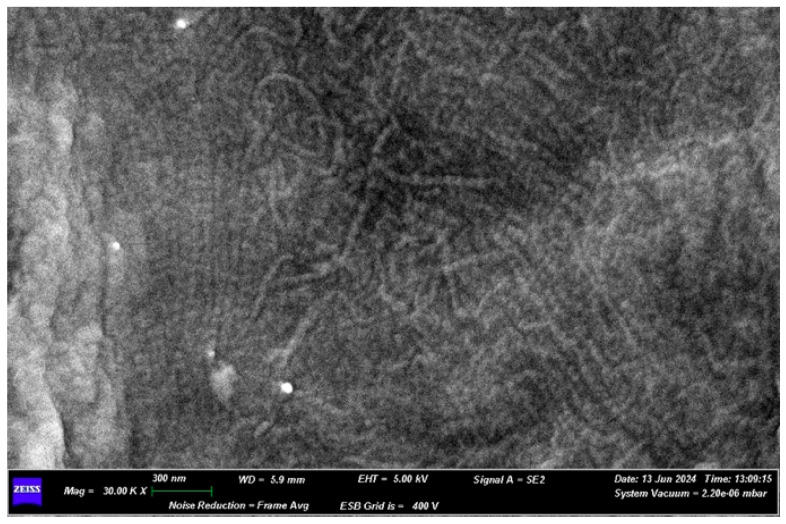
30.00K× SEM magnification of the surface A.

In detail of surface A, observed under 30.00K× magnification, it is possible to note the dense network of collagen fibers that constitute the surface of the membrane (Figure 13). The fibers have variable dimensions: 50 nm, 33 nm and 67 nm.

**Figure 14:**
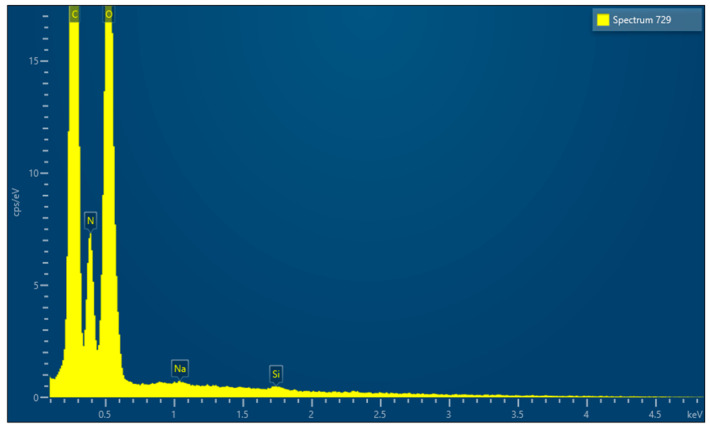
EDX results.

EDX microanalysis performed by scanning electron microscope showed the chemical elements constituting the membrane: C, N, O, Na and Si (Figure 14).

Finally, after cutting the membranes to obtain a square-shaped sample, measuring 1 cm × 1 cm, the mass of the samples were calculated, which is equal to 34.2 (0.5) mg. After obtaining the volume of the samples (V = a × b × c = 1 cm × 1 cm × 0.0436 cm = 0.0436 cm^3^), we calculated their main density (d = m/V), which is equal to 7.844 (0.6) g/cm^3^.

### 3.4. Sample 4

#### 3.4.1. Commercial Information

Commercial information Straumann^®^ Membrane Flex is a minimally cross-linked porcine peritoneal collagen membrane (types I and III). Minimal aldehyde cross-linking provides intrinsic strength and predictable absorption over 12–16 weeks while preserving handling characteristics. Manufacturing includes purification to remove non-collagenous components, low-concentration aldehyde cross-linking, thorough rinsing in demineralised water, drying, sizing, double-pouch packaging and gamma sterilization. The membrane can be applied dry or hydrated (≈5 min in sterile water or saline). It may be cut to size wet or dry and placed with either side facing the bone. An overlap of ≥2 mm beyond defect walls is recommended; fixation with absorbable sutures may be used. Sizes: 15 × 20 mm, 20 × 30 mm, 30 × 40 mm.

#### 3.4.2. Optical Microscope and Scanning Electron Microscope Analysis

**Figure 15:**
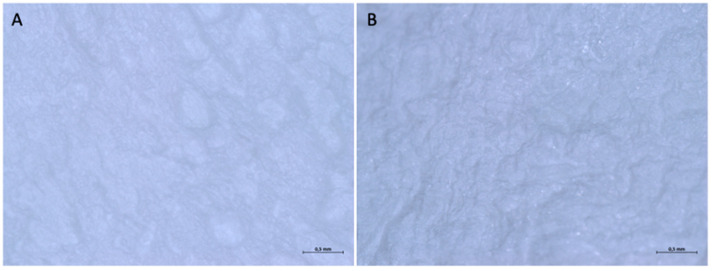
(**A**) Optical micrograph of Surface A at 32× magnification. (**B**) Optical micrograph of Surface B at 32× magnification.

When examined under an optical microscope, both at 16× magnification and at a higher magnification of 32×, it can be seen that both surfaces are rough, due to irregular depressions in the surface that generate a texture with random geometry (Figure 15).

**Figure 16:**
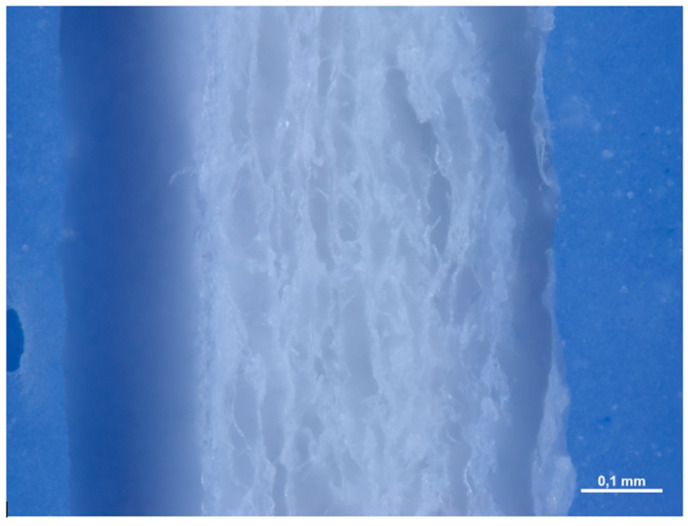
Cross-sectional image of the membrane at 168× magnification under the optical microscope.

In section, it is not possible to observe a precise stratification, since the material appears to be made up of an interwoven network of fibers without a precise organization (Figure 16). The main thickness of the membrane is equal to 0.491 (0.03) mm.

When examined with a scanning electron microscope (SEM), at a first observation with a magnification of 200×, the two surfaces do not show substantial differences. Both sides of the membrane have a compact appearance, with an irregular surface due to the presence of ridges and depressions.

**Figure 17:**
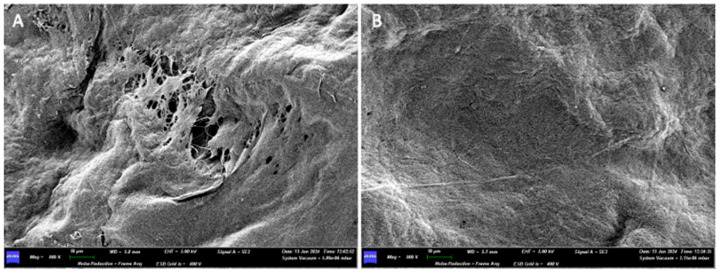
(**A**) 800× SEM magnification of the surface A. (**B**) 800× SEM magnification of the surface B.

At 800× magnification, while a network of collagen fibers can be seen on surface A, surface B still appears compact, and no collagen fibers can be appreciated there (Figure 17).

Side B of the membrane, even when increasing the magnification (1.50K×), presents a very compact and homogeneous surface, in which neither collagen fibers nor pores between the fibers themselves are appreciable.

**Figure 18:**
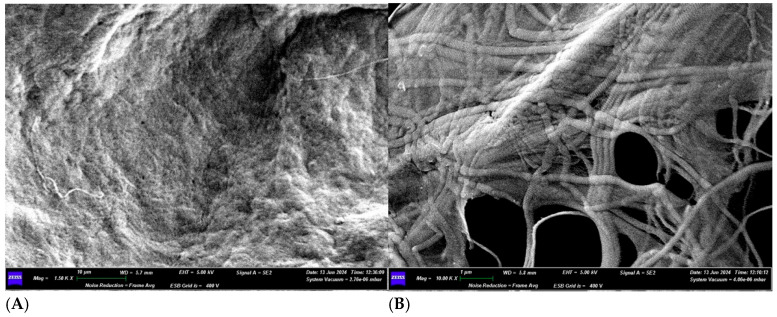
(**A**) SEM image of Surface B at 1500× magnification. (**B**) SEM image of Surface A at 10,000× magnification.

On the other hand, when increasing the magnification (10.00K×) on side A of the membrane, we can observe how multiple fibers join together in bundles, interwoven with each other (Figure 18). Some fibers run perpendicularly to bundles made up of parallel fibers. The thicknesses of some fibers are 0.048 μm, 0.095 μm, 0.150 μm, 0.200 μm, and 0.250 μm. The thickness of a bundle made up of four fibers is 0.700 μm.

**Figure 19:**
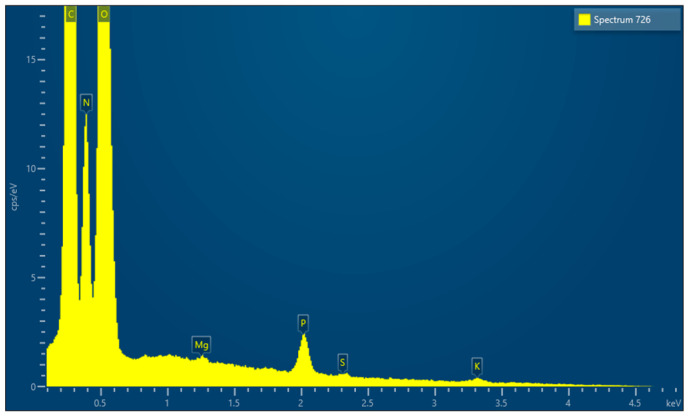
EDX results.

EDX microanalysis performed by scanning electron microscope showed the chemical elements constituting the membrane: C, N, O, Mg, P, S and K (Figure 19).

Finally, after cutting the membranes to obtain a square-shaped sample, measuring 1 cm × 1 cm, the mass of the sample was calculated, which is 16.0 (2.78) mg. After obtaining the volume of the samples (V = a × b × c = 1 cm × 1 cm × 0.0491 cm = 0.0491 cm^3^), we calculated their main density (d = m/V), which is 0.326 (0.01) g/cm^3^.

### 3.5. Sample 5

#### 3.5.1. Commercial Information

Commercial information XC Collagen^®^ Xenomatrix is a three-dimensional collagen matrix manufactured from equine-tendon type I collagen. It can be used alone or with suitable graft materials to protect grafted sites from soft-tissue and epithelial invasion. The barrier effect lasts 2–4 weeks, after which endogenous collagenases initiate resorption. Xenomatrix may be left exposed only in alveolar-preservation procedures. If required, shape to fit the defect; do not hydrate—the device must be applied dry. Indications include post-extraction alveolar preservation and Miller Class I–II gingival recession. For sockets, the BCG-XC10 format is recommended (one octagonal and one circular patch). Sterilization is by beta irradiation at 25 kGy. Dimensions: 38 × 16 × 4/Ø14 × 4 mm.

#### 3.5.2. Optical Microscope and Scanning Electron Microscope Analysis

When examined under an optical microscope, with a 16× magnification, it can be seen that the two surfaces of the membrane appear different. Surface A is smoother and more homogeneous, although it still has a microtexture consisting of parallel curvilinear striae, which go from the lower right corner to the upper left corner, with a sigmoidal appearance. Surface B is rougher and more jagged, with a random pattern.

**Figure 20:**
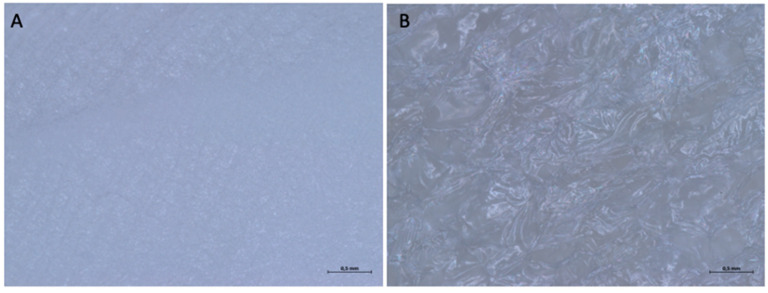
(**A**) 32× magnification under optical microscope of the surface A. (**B**) 32× magnification under optical microscope of the Surface B.

At a higher magnification, we can observe how the distance between the striae, which makes up the microtexture of Surface A, is in the order of microns (Figure 20). Surface B has a shiny appearance, almost as if it were covered with a film.

**Figure 21:**
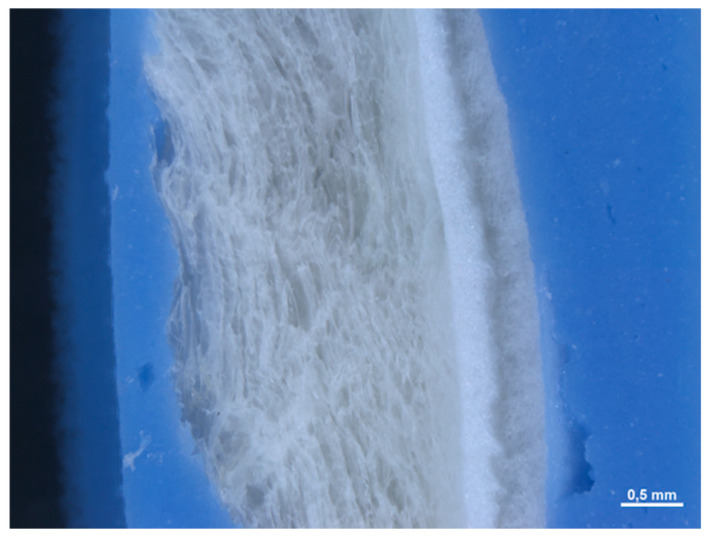
Cross-sectional image of the membrane at 25× magnification under the optical microscope.

In section, we observe how the membrane is made up of different layers (Figure 21). We have two layers made up of a dense woven network on the two sides of the matrix (with a thickness of 2.309 mm and 0.357 mm), divided by a spongy-like layer (with a thickness of 0.333 mm).

When examined under a scanning electron microscope (SEM) at 30× magnification, the two sides of the membrane appear different. Surface A appears smoother, while Surface B is more porous and alveolized.

At 400× magnification, on side A of the membrane, an unordered geometry can be observed, resembling multiple sheets stacked on top of each other but without a precise pattern. On side B, a single sheet that makes up the membrane can be appreciated.

**Figure 22:**
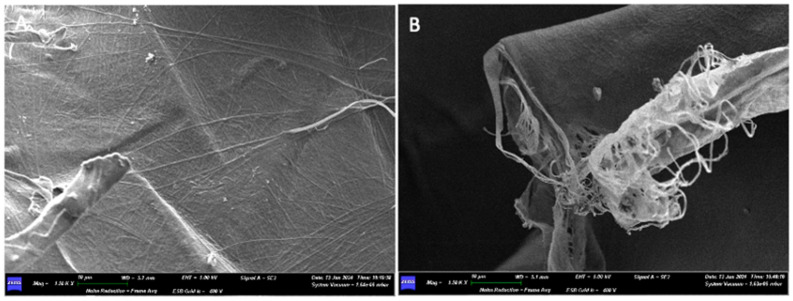
(**A**) SEM image of Surface A at 1500× magnification. (**B**) SEM image of Surface B at 800× magnification.

Increasing the magnification further, at 1.50K×, on side A, the individual collagen fibers that make up the membrane can be appreciated; however, they are highly compacted together and are likely embedded in a matrix that holds them all together, forming a sheet. On side B, it is possible to see how the sheet is composed of individual fibers, thanks to the detail showing the fraying of the sheet itself (Figure 22).

In section, the matrix has a structure made up of multiple parallel layers, joined by fibers perpendicular to them. This structure forms pores, determining a honeycomb structure, reminiscent of a bees’ nest. The pores have a variable geometric shape, some predominantly square or rectangular, others irregular. The distance between the various layers is variable: 0.221 mm, 0.158 mm, 0.079 mm, 0.284 mm. The dimensions of the more regularly shaped pores are also variable: 0.217 × 0.200 mm, 0.450 × 0.183 mm, 0.333 × 0.100 mm.

At 400× magnification, the individual layers that constitute it can also be seen, which define pores of varying sizes: 27.50 × 37.50 μm, 30.00 × 22.50 μm, 5.00 × 3.75 μm.

**Figure 23:**
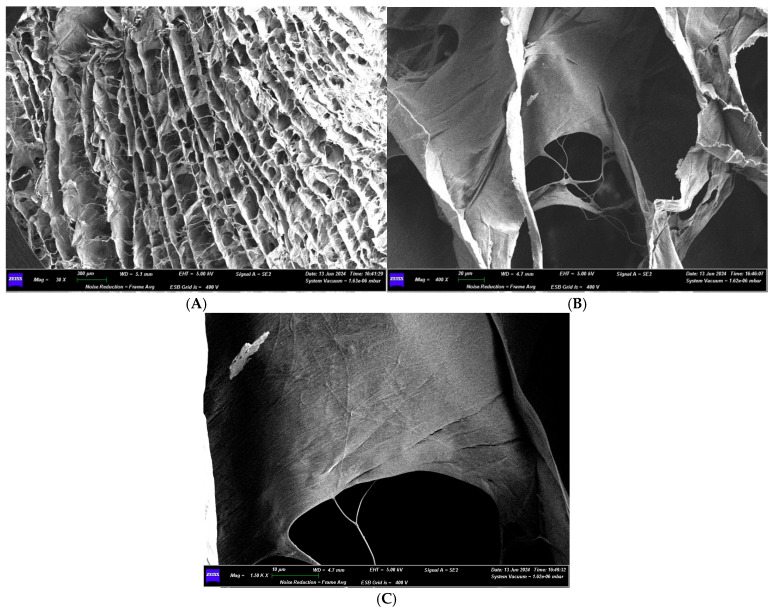
(**A**) SEM image of the membrane section at 30× magnification. (**B**) SEM image of the membrane section at 400× magnification. (**C**) SEM image of the membrane section at 1500× magnification.

Increasing the magnification further, at 1.50K×, the sheet, composed of compacted collagen fibers and pores, can be appreciated. The fibers have a thickness of about 0.312 μm (Figure 23).

**Figure 24:**
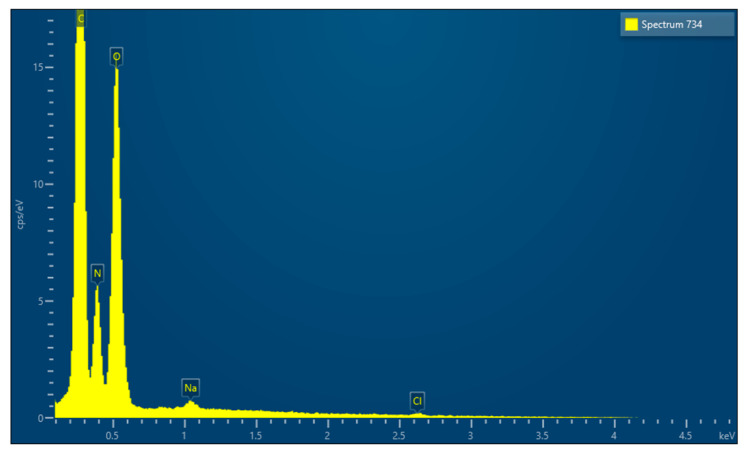
EDX results.

EDX microanalysis performed by scanning electron microscope showed the chemical elements constituting the membrane: C, N, O, Na and Cl (Figure 24).

Finally, after cutting the membrane to obtain a square-shaped sample measuring 1 cm × 1 cm, the mass of the sample was calculated to be 16.1 (2.78) mg. After obtaining the volume of the samples (V = a × b × c = 1 cm × 1 cm × 0.2941 cm = 0.2941 cm^3^), we calculated their main density (d = m/V), which is 0.055 (0.006) g/cm^3^.

The main results are presented in Table 1.

## 4. Discussion

As we know from studying other implantable medical devices such as endosseous implants or bone substitutes, it is crucial to know the macro- and micromorphology of the membrane as it can influence healing patterns. This paper is the first describing macro and micromorphology of Sample 3 and Sample 5.

At the macroscopic level, the presence of depressions and reliefs gives all the membranes a certain roughness and texture on both sides. This can be appreciated both with the naked eye and under an optical microscope. However, when increasing magnification with a scanning electron microscope (SEM), we notice that not all the membranes present the same degree of roughness and the same microscopic structure on both surfaces, sometimes even contrary to what is specified in the leaflet by the manufacturer itself.

The Sample 1 membrane is described in the leaflet as having a two-layer structure: the rough surface, in contact with the bone, favors the attachment of osteogenic cells; the smoother surface, in contact with soft tissues, prevents the infiltration of fibrous tissue into the bone defect. Optical and SEM analysis confirmed the structural difference between the two sides of the membrane. Additionally, the presence of collagen fibers is clearly visible. It is one of the most investigated membranes in the literature and widely used in bone regeneration procedures. It has a density and trabeculation such that periosteal perfusion is not hindered and being non-crosslinked an excellent biocompatibility.

One issue might be degradation times that may not be long enough especially if exposures happen.

For the Sample 2 membrane, the manufacturer does not specify which side should come into contact with the clot and which with soft tissues. Optical and SEM analysis revealed no structural differences between the two sides of the membrane. This explains why the instructions for use do not specify how to position the membrane: since both sides are the same, they can be placed indiscriminately in contact with either the bone or soft tissues. Furthermore, the SEM analysis allowed us to clearly observe the dense network of collagen fibers that constitute the membrane. Once again, the focus is on a membrane that is both widely studied and widely used. The cross-linking process by glycation allows for increased resistance to degradation while maintaining excellent biocompatibility. The network defines a labyrinth of pores of varying sizes, confirming the presence of a porous structure described in the leaflet. Some fibers are single, while others are intertwined and twisted together. These differences create a different permeability (the property of certain substances to allow liquids or gases to pass through or penetrate).

Sample 3 is different from the other materials under study, as it is a sheet of bone (equine origin) collagenated, deantigenated, and partially demineralized. The manufacturer does not specify which side of the membrane should come into contact with the bone, implying that both sides can be in contact with either the bone or soft tissues. However, there are substantial differences between the two sides of the membrane. Under optical microscope analysis, both sides show a rough surface, but with two different patterns: one surface has a regular texture with horizontal parallel striations, creating a striped effect, while the other shows roughness generated by irregularly distributed depression areas throughout the surface. Upon SEM analysis, one side is smoother, more compact, and regular, while the other is more irregular due to pores and depressions of varying shapes and sizes. Both sides display collagen fibers, which are compact and adhere to the surface on the smoother side, while free fibers are visible on the other side. Both sides of the membrane show pores that define canalicular structures resembling bone canaliculi. This suggests that despite the treatment it underwent, the bone sheet has maintained its typical three-dimensional structure. These types of barriers have an exceptional barrier effect and provide mechanical support, but at the same time they partially inhibit external perfusion. They are more suitable in cases of horizontal containment defects where there are three bone walls that can provide biological support for regeneration.

The Sample 4, according to the manufacturer’s instructions, does not have a specific side, and both sides can be in contact with the bone. Optical microscope analysis shows that the two surfaces are almost identical: both are rough due to irregular depressions on the surface that generate a pattern with random geometry. However, SEM analysis revealed a structural difference between the two sides of the membrane. While one side shows a dense network of collagen fibers, intricately intertwined, the other appears more compact and homogeneous, and no clear collagen fibers are visible. There is little scientific data available on this recently introduced membrane. Aldehyde cross-linking gives it considerable resistance to degradation when not exposed, and careful pre-packaging rinsing procedures ensure that its cytotoxicity due to aldehydes is minimized.

Sample 5, which is a matrix and not a collagen membrane, does not specify in the leaflet the presence of a specific side. However, both optical and SEM analysis showed differences between the two surfaces of the matrix. While both have micro-roughness, one surface is smoother and more compact, while the other is more jagged and irregular. Furthermore, SEM cross-section analysis reveals a porous structure consisting of several parallel layers, connected by perpendicular fibers. This structure creates a network of pores resembling a honeycomb. Both sides of the membrane display collagen fibers: on the smoother side, the collagen fibers are highly compacted together and likely immersed in a matrix that holds them together to form a sheet; on the more jagged side; the sheet is made up of individual fibers, as evidenced by the detail of the sheet’s fibrillation. Its matrix texture gives it an important thickness but with the lowest density. This structure certainly promotes periosteal perfusion, but its degradation times may not be sufficient to ensure proper bone regeneration. These hypotheses should be confirmed by scientific studies on this matrix that are absent to date.

It is interesting to note that among the membranes for which no specific side is indicated for placement in contact with the bone, only Sample 2 shows no differences between the two sides at both the macroscopic and microscopic levels. The others (Sample 3, Sample 4, Sample 5) show substantial differences between the two sides, and thus there may be a difference in their potential regenerative capacity. Further studies, both in vitro and in vivo, are needed to explore this topic in more depth.

In cross-section, under optical microscope analysis, Sample 3 is the only membrane that shows a compact surface where the only voids are pores that strongly resemble bone canaliculi, and within the membrane, they will likely create a canalicular network for the passage of vessels, blood, and nutrients to the newly formed bone. Sample 2 is the only one that shows a structure formed by several layers compacted together in cross-section. The other membranes (Sample 1, Sample 4, Sample 5) show a structure consisting of a dense network of intertwined fibers, creating a porous structure. The different cross-sectional structures of the various membranes also affect the permeability of the membranes themselves, and thus the ability to allow liquids, such as blood, to pass through or penetrate. This may impact the potential regenerative capacity of the scaffolds.

When examined with a scanning electron microscope, the only membrane to show an interesting structure in cross-section from a permeability perspective is Sample 5. It features a structure resembling a honeycomb, made up of several parallel layers, connected by perpendicular fibers. The pores vary in shape, with some being mostly square or rectangular and others irregular. This dense porous network allows the free passage of fluids within it.

When comparing the collagen fibers that make up the various membranes under study, no substantial differences are observed in their structure and size. In fact, the thickness of the fibers varies across all materials from about 0.050 μm to 0.300 μm. Additionally, in all the membranes, the fibers can either be free or intertwined and twisted together, creating a dense network. This also affects the permeability of the material: the more the fibers are intertwined and compacted, the less the membrane will allow fluids, such as blood, to pass through. Finally, no substantial differences are observed between collagen from porcine and equine origin: their three-dimensional structure and size are nearly identical.

EDX, conducted using a scanning electron microscope, revealed that all the membranes consist of three common elements (C, N, and O) and minor components that vary from membrane to membrane.

Each barrier membrane was cut into square samples, measuring 1 × 1 cm, and the mass and density were calculated. The heaviest membrane was Sample 3 (34.2 mg), and the lightest was Sample 2. The weight difference between the analyzed samples obviously varies based on the composition of the membranes: collagen-based ones are lighter than those made from bone sheets. Sample 4 and Sample 5 both have the same mass (16.0–16.1 mg): it is interesting to note that despite having the same mass, their thickness varies (0.491–2.941 mm). Similarly, Sample 1 and Sample 4 have almost the same thickness (0.471–0.491 mm), but the former has a lower mass than the latter (9.7–16.0 mg). At this point, we calculated the density of all the samples, relating the mass (in g) to the volume (in cm^3^).

Observing the results presented in Table 1, it is interesting to note how, for membranes with the same mass but different thickness, or the same thickness but different mass, the density of the membranes changes significantly. This translates into a different degree of material compaction and a varying permeability of the membranes. In fact, the material with the lowest density is Sample 5, which, when examined in cross-section with SEM, displayed a honeycomb-like porous structure. As permeability increases, the potential regenerative capacity of the material also increases, as the membrane will allow blood to pass through more easily. It is necessary to confirm this hypothesis through in vivo studies.

The type of cross-linking and the subsequent procedures before packing the membrane are also decisive in defining its biocompatibility. As a recent article report, the cross-linked membrane prolong their degradation up to 12 weeks instead those in native collagen already show signs of advanced degradation at 4 weeks [26].

A clinical study comparing cross-linked membrane (CLM) with native collagen membrane (CM) shows higher perforation frequency in CLM; one of the reasons could be the inverse correlation between the grade of cross-linking and the early angiogenesis [21]. The same study observing the vascularization of the Sample 1 membrane reports that the biocompatibility and porosity of native collagen is crucial to allow external vascularization from the periosteum. Nine years later, these findings were confirmed on a very accurate histological study that shows neoangiogenesis and peripherical bone formation through the native collagen membrane at 30 days post-op [16].

Only one previous study describes Sample 4 by reporting SEM analysis, suture tensile strength and in vivo animal implantation histologies comparing them with Sample 1 ones. Microscopic analyses show similar results to the present study: no difference between the two sides and a denser internal reticulation than Sample 1. The suture test reports three times the strength of the native collagen membrane, which can be explained by the denser structure. Furthermore, biopsies at 4, 8 and 12 weeks show that Sample 4 is more resistant to degradation but at the same time more biocompatible. Biopsies in rabbits show a lower concentration of inflammatory infiltrate at 4 and 8 weeks; this can be explained by the minimal presence of cross-linking and the generous rinsing procedure that does not react molecules and other cytotoxic residues of the aldehyde reticulation process [19,23].

Sample 1 is the most studied in the literature, while the other samples have great potential but need further research to assess how the differences between the two sides of the membrane can influence cell adhesion and proliferation and angiogenesis, especially during the early stages of healing.

It should be noted that there are some limitations that may undermine the inferences of this study, such as the absence of experiments using bone-lineage cells that could confirm both the rate of colonization and the rate of degradation of the membrane itself.

Future articles incorporating osteogenic cells could clarify how specific microstructural features influence regenerative progression.

## 5. Conclusions

Although macroscopically similar, the membranes exhibit distinct microscopic and structural differences that may influence bone regeneration and socket stability.

Densities vary widely, reflecting different degrees of material compaction and, by inference, permeability. Greater permeability may enhance regenerative capacity by facilitating blood passage, while biocompatibility governs cellular colonization. Membrane choice should be tailored to the clinical situation, prioritizing properties most advantageous for the bone defect.

## Figures and Tables

**Table 1:** Mass, thickness and density of the five membranes under study.

Barrier Membrane	Mass	Thickness	Density	Structure	Chemical Composition
Sample 1	9.7 (0.2) mg	0.471 (0.041) mm	0.206 (0.02) g/cm^3^	Native collagen	C, N, O
Sample 2	6.6 (0.13) mg	0.245 (0.014) mm	0.269 (0.04) g/cm^3^	Crosslinked	C, N, O, Na, Cl, P
Sample 3	34.2 (0.5) mg	0.436 (0.044) mm	7.844 (0.6) g/cm^3^	Bone	C, N, O, Na, Cl
Sample 4	16.0 (0.20) mg	0.491 (0.03) mm	0.326 (0.01) g/cm^3^	Crosslinked	C, N, O, Mg, P, S, K
Sample 5	16.1 (2.78) mg	2.941 (0.) mm	0.055 (0.006) g/cm^3^	Native Collagene	C, N, O, Na, Si

## Data Availability

The data are contained within the article.
